# Comparative Study of Brain fMRI of Olfactory Stimulation in Neuromyelitis Optica Spectrum Disease and Multiple Sclerosis

**DOI:** 10.3389/fnins.2021.813157

**Published:** 2022-01-10

**Authors:** Shaoyue He, Tingting Peng, Weiwei He, Chen Gou, Changyue Hou, Juan Tan, Xiaoming Wang

**Affiliations:** ^1^Institute of Neurological Diseases, North Sichuan Medical College, Nanchong, China; ^2^Department of Neurology, The People’s Hospital of Dazu, Chongqing, China; ^3^Department of Neurology, Affiliated Hospital of North Sichuan Medical College, Nanchong, China

**Keywords:** neuromyelitis optica spectrum disease, multiple sclerosis, olfactory, functional magnetic resonance imaging, brain

## Abstract

**Objective:** To observe the characteristics of brain fMRI during olfactory stimulation in patients with neuromyelitis optica spectrum disease (NMOSD) and multiple sclerosis (MS), compare the differences of brain functional activation areas between patients with NMOSD and MS, and explore the characteristics of olfactory-related brain networks of NMOSD and MS.

**Methods:** Nineteen patients with NMOSD and 16 patients with MS who met the diagnostic criteria were recruited, and 19 healthy controls matched by sex and age were recruited. The olfactory function of all participants was assessed using the visual analog scale (VAS). Olfactory stimulation was alternately performed using a volatile body (lavender and rose solution) and the difference in brain activation was evaluated by task-taste fMRI scanning simultaneously.

**Results:** Activation intensity was weaker in the NMOSD group than in the healthy controls, including the left rectus, right superior temporal gyrus, and left cuneus. The activation intensity was stronger for the NMOSD than the controls in the left insula and left middle frontal gyrus (*P* < 0.05). Activation intensity was weaker in the MS group than the healthy controls in the bilateral hippocampus, right parahippocampal gyrus, right insula, left rectus gyrus, and right precentral gyrus, and stronger in the left paracentral lobule among the MS than the controls (*P* < 0.05). Compared with the MS group, activation intensity in the NMOSD group was weaker in the right superior temporal gyrus and left paracentral lobule, while it was stronger among the NMOSD group in the bilateral insula, bilateral hippocampus, bilateral parahippocampal gyrus, left inferior orbital gyrus, left superior temporal gyrus, left putamen, and left middle frontal gyrus (*P* < 0.05).

**Conclusion:** Olfactory-related brain networks are altered in both patients, and there are differences between their olfactory-related brain networks. It may provide a new reference index for the clinical differentiation and disease evaluation of NMOSD and MS. Moreover, further studies are needed.

## Introduction

Neuromyelitis optica (NMO) is an idiopathic inflammatory demyelinating disease of the central nervous system; it mainly affects the optic nerve and spinal cord and usually does not affect the brain (Wingerchuk et al., 2007). Multiple sclerosis (MS) is an autoimmune disease of the central nervous system, most commonly involving the paracortex, periventricular, optic nerve, spinal cord, brainstem, and cerebellum (Brownlee et al., 2017). NMO was previously considered to be a subtype of MS. After Wingerchuk et al. (1999) proposed the diagnostic criteria of NMO, Lennon et al. (2004) first described an autoantibody with high specificity for NMO named NMO-IgG. NMO-IgG was identified as an aquaporin-4 antibody (AQP4-Ab) that selectively binds to the aquaporin-4 (AQP4) (Lennon et al., 2005). AQP4-IgG provides a means to distinguish NMO from MS (Wingerchuk et al., 2006) and helps define neuromyelitis optica spectrum disorders (NMOSD). Optic neuritis, acute myelitis, and area postrema syndrome are the most common clinical symptoms of NMOSD. In addition, there are three groups of clinical symptoms: acute encephalic syndrome, acute diencephalic syndrome, and encephalic syndrome (Wingerchuk et al., 2015; Fujihara, 2019). Previous research indicates that patients with NMOSD and MS may experience cognitive impairment (Oertel et al., 2019) and olfactory dysfunction (Zhang et al., 2015), in addition to manifestations of the optic nerve, spinal cord, and brain involvement. Olfactory disorders have been described in a variety of neurodegenerative diseases (Chen et al., 2014; Das, 2021), such as Parkinson’s disease (PD), Alzheimer’s disease (AD), and multiple system atrophy. MS olfactory disorder has attracted wide attention from scholars worldwide, but there are few studies on NMOSD olfactory disorders (Schmidt et al., 2013; Joseph and DeLuca, 2016).

The mechanism of olfactory disorders remains unclear. Demyelination is a common pathological feature of both diseases (DeLuca et al., 2015). Some studies indicate that demyelination changes occur in the olfactory bulb/olfactory tract in both patients with NMO and with MS, but there are still differences between the two diseases (DeLuca et al., 2015). AQP4 is strongly expressed in the synaptic units of the olfactory bulb. Schmidt et al. (2013) observed that AQP4-IgG tightly binds to the olfactory bulb of rats and mice. DeLuca et al. (2015) demonstrated a selective loss of AQP4 in the olfactory bulb/olfactory tract injury in patients with NMO. The above studies suggest that AQP4-IgG could cause tissue damage in the olfactory structures expressing AQP4, causing olfactory disorders. In NMOSD, the olfactory detection threshold is positively correlated with AQP4-IgG levels (Zhang et al., 2015). Olfactory-related brain damage in MS may be caused by systemic immune T cells infiltrating the submembranous space through fluid circulation and then migrating to the brain parenchyma through the periductal space (Shin et al., 2019).

Olfactory dysfunction is present in both patients with NMOSD and with MS, with incidence rates of 11–50% (Joseph and DeLuca, 2016) and 50–53%, respectively (Schmidt et al., 2013; Zhang et al., 2015). Schmidt et al. (2013) observed olfactory recognition and discrimination disorders in 50% of patients with NMO using the Sniffin’ sticks olfactory test. Olfactory detection threshold and recognition threshold in patients with NMOSD are negatively correlated with olfactory bulb volume (Zhang et al., 2015). Dysosmia appears in the early stage of MS (Rolet et al., 2013), and the threshold of olfactory detection increases significantly during the active stage of MS inflammation (Lutterotti et al., 2011), which is related to the peripheral olfactory system, and the impairment of olfactory discrimination is more obvious in MS patients with a chronic disease course. Olfactory recognition and identification functions are considered to be related to the brain regions involved in olfactory processing. Olfactory processing involves different regions of the brain, including the piriform layer, amygdala, insula, orbitofrontal cortex, cingulate, and thalamus (Ciorba et al., 2020). Doty et al. (1999) observed a negative correlation between UPSIT scores and the number of plaques in the inferior frontal and temporal lobes in patients with MS. Olfactory-related imaging studies have found that olfactory dysfunction is associated with olfactory bulb volume loss in patients with MS (Goektas et al., 2011). Recent evidence indicates that olfactory bulb volume reduction also exists in patients with NMOSD olfactory disorders (Zhang et al., 2015). Li et al. (2018) compared NMO and MS patients with olfactory disorders and demonstrated that olfactory bulb volume and right orbitofrontal lobe volume in patients with NMO were significantly lower than those in patients with MS. Olfactory disorders in patients with NMOSD and with MS are associated with olfactory damage, but the mechanism of olfactory disorders remains unclear for both. Improved understanding of olfactory-related structure and function will assist better in distinguishing NMOSD from MS.

Functional magnetic resonance imaging (fMRI) is an oxygen-dependent technique that can objectively detect the activity of olfactory brain regions through the localization of flow changes and metabolic changes in the brain during neuronal activity and adult brain function images. In this study, we used fMRI combined with an olfactory event-related design to observe the characteristics of olfactory-related brain networks and the differences between NMOSD and MS patients and to provide a new reference index for clinical differentiation and disease evaluation of NMOSD and MS.

## Materials and Methods

### Participants

Nineteen patients with NMOSD were recruited from the Department of Neurology of the Affiliated Hospital of North Sichuan Medical College from July 2019 to December 2020. Two patients were excluded due to head movement during fMRI acquisition; thus, 17 patients were enrolled in the study. Sixteen patients with MS were enrolled in the same period, and 19 healthy adults matched by sex, age, and education were recruited as the control group. Inclusion criteria entailed: (1) clinical manifestations of NMOSD according to 2015 international NMOSD diagnostic criteria, clinical manifestations of MS according to 2010 version of McDonald diagnostic criteria; (2) no severe medical diseases and able to cooperate to complete the study; (3) routine MRI scan did not reveal other intracranial organic lesions, such as stroke or brain tumors; and (4) signed informed consent.

We excluded patients according to the following criteria: (1) recently had an acute upper respiratory tract infection, antrum, or sinus diseases; (2) combined with other diseases affecting olfactory function including AD, PD, anxiety, depression, or schizophrenia; (3) had a history of smoking or alcohol abuse; or recent using of drugs that may lead to olfactory disorders, such as glucocorticoids, atriptyline, or D-clozymide.

This study followed the ethical principles of the Declaration of Helsinki. It is approved by the Ethics Committee of the Affiliated Hospital of North Sichuan Medical College (approval no. 2021ER004-1). Informed consent was signed by all participants.

### Clinical Data Collection

Clinical information, course of disease, and EDSS score were collected, and the olfactory function of all participants was evaluated using the VAS scale (Gupta et al., 2014) (0 for complete loss of sense of smell and 5 for a completely normal sense of smell).

### Olfactory Stimulation Method

Emerging Tech Trans (ETT) olfactory stimulator (Hershey Company, United States) was selected. The preset olfactory stimulation paradigm was used. The volatile body (0.5% fumigation grass, 0.5% rose solution) was selected to alternately enter olfactory stimulation to avoid olfactory adaptation. The ETT olfactory stimulator can accurately control the flow and concentration of the smell source and release it at fixed time intervals so that olfactory stimulation can be accurately repeated in a short time.

### Functional Magnetic Resonance Imaging Data Acquisition

The MRI data of all participants were collected with a 3.0 T superconducting MR (DISCOVERYMR750, GE, United States). We used a 32-channel phased-array head coil. During the data collection, participants were asked to relax their entire body, rest quietly, and remain awake. If unbearable discomfort occurred during the examination, scanning was stopped.

Advanced conventional MRI scanning, including T1WI and T2WI, then started the ETT olfactory stimulator. According to the preset stimulation paradigm, the participants were stimulated by olfactory stimulation, and the task-state fMRI scanning was synchronized. fMRI scanning parameters were as follows: echoplanar imaging (EPI) technology, TR = 2,000 ms, TE = 30 ms, flip angle = 90°, slice thickness = 4 mm, scanning slices = 33, matrix = 64 × 64, and field of view = 24 × 24. Olfactory stimulation paradigm: First, 42 s of clean air (3 L/min) was given as the stimulation interval, then 6 s of stimulation (3 L/min, alternating rose and fumigation) was provided and repeated 12 times.

### Data Processing and Analysis

Statistical analyses were performed using SPSS 23.0. Normally distributed data are expressed as “mean ± standard deviation.” Age, years of education, and VAS scores were analyzed by one-way Analysis of variance (ANOVA); sex was analyzed by card test. EDSS scores and disease course were analyzed using a two-sample *t*-test.

SPM8 and Metlab2013a were used to preprocess the fMRI images of each participant, including time correction, head movement correction, spatial standardization, and spatial smoothing. The images of the first five time points were removed to ensure the stability of longitudinal magnetization at the beginning of the scanning. The activation of brain regions in the intra-group among three groups was analyzed using a single-sample *t*-test. The difference in activated brain regions between the three groups was analyzed by one-way ANOVA, then *Post Hoc* pairwise comparisons were performed with the Tukey-Kramer test to analyze the difference between each two groups; All fMRI data analysis are corrected using AlphaSim, and *P* < 0.05, indicating that the difference was statistically significant. Brain regions with activated cluster values of > 10 voxels indicated meaningfully activated brain regions.

## Results

### Demographic and Clinical Characteristics

Seventeen patients (14 females, 3 males) with NMOSD were enrolled, with an average age of 46.65 (± 13.29) (range 22–66) years, disease duration of 4.05 (± 1.86) years, including 15 AQP4-IgG positive cases and 2 AQP4-IgG negative cases. There were 16 patients (12 females, 4 males) with MS, with an average age of 44.38 (± 14.59) (range 25–69) years, and disease duration of 5.20 (± 5.21) years. There were 19 healthy controls (13 females, 6 males), with an average age of 40.47 (± 9.35) (range 28–60) years. There were no significant differences in age, sex, or years of education among the three groups (*P* > 0.05). There was no significant difference in the disease duration between the NMOSD and MS groups (*P* > 0.05). There were significant differences in VAS scores between the NMOSD and control groups or between the MS and control groups (*P* < 0.001). There was no significant difference in the olfactory VAS scores between the NMOSD and MS groups (*P* > 0.001) (Table 1).

**TABLE 1:** Comparison of statistical and clinical data.

Items	NMOSD group	MS group	Control group	*P-*values
Sex (male/female)	17 (3/14)	16 (4/12)	19 (6/13)	0.614
age (years)	46.65 ± 13.29	44.38 ± 14.59	40.47 ± 9.35	0.329
Years of education (years)	8.53 ± 3.79	8.75 ± 4.69	9.53 ± 3.53	0.735
Disease course (years)	4.05 ± 1.86	5.20 ± 5.21		0.417
VAS score	2.79 ± 0.47*	2.84 ± 0.50*	4.47 ± 0.46	0.000
EDSS score (points)	3.47 ± 1.69	3.12 ± 2.65		0.657
AQP4-IgG (±)	17 (15/2)			

NMOSD, neuromyelitis optica spectrum disease; MS, multiple sclerosis; VAS, Visual Analog Scale; EDSS, Extended Disability Status Scale; AQP4-IgG, aquaporin-4 waterchannel–IgG; *, no statistically significant between-group difference.

### Whole-Brain Activation Distribution

#### Intra-Group Analysis of Whole-Brain Activation

**FIGURE 1:**
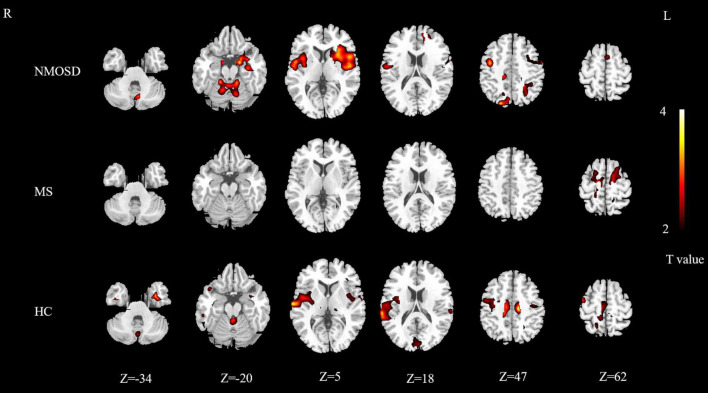
Distribution map of activated brain regions in NMOSD group, MS group, and control group. Yellow to red indicates strong to weak activation. NMOSD, neuromyelitis optica spectrum disease; MS, multiple sclerosis; HC, healthy controls.

The areas activated by olfactory stimulation in the control group included the bilateral insula, bilateral superior parietal gyrus, bilateral supplementary motor area, bilateral superior temporal gyrus, bilateral cuneus, bilateral paracentral lobule, bilateral medial cingulate gyrus, right precentral gyrus, left inferior temporal gyrus, right rolandic operculum, right supramarginal gyrus, left fusiform gyrus, right putamen, right middle frontal gyrus, right superior occipital gyrus, and left cerebellum (*P* < 0.05; Figure 1).

The activated brain areas in the MS group included the bilateral precentral gyrus, bilateral dorsolateral superior frontal gyrus, bilateral supplementary motor area, left paracentral lobule, and left lingual gyrus (*P* < 0.05; Figure 1).

The activated brain areas in the NMOSD group included the bilateral insula, bilateral amygdala, bilateral hippocampus, bilateral rolandic operculum, bilateral parahippocampal gyrus, bilateral precentral gyrus, bilateral posterior central gyrus, bilateral putamen, bilateral superior temporal gyrus, bilateral cerebellum, bilateral Heschl’s gyrus, left orbital inferior frontal gyrus, left triangle inferior frontal gyrus, left opercular part of inferior frontal gyrus, left caudate nucleus, left middle frontal gyrus, left inferior temporal gyrus, left pallidum, right lingual gyrus, left fusiform gyrus, left inferior occipital gyrus, left dorsolateral superior frontal gyrus, left medial superior frontal gyrus, right superior occipital gyrus, right precuneus, right cuneus, left superior parietal cortex, left parietal inferior lobe angular gyrus, right medial cingulate cortex, left supplementary motor area (*P* < 0.05; Figure 1).

#### Between-Group Differences in Whole-Brain Activation

**FIGURE 2:**
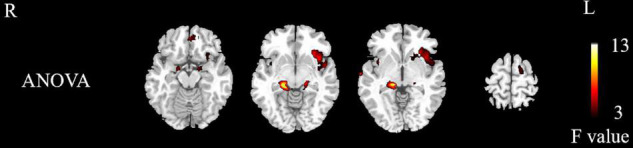
Differences in activated brain regions between NMOSD group, MS group, and control group. Yellow to red indicates strong to weak activation. NMOSD, neuromyelitis optica spectrum disease; MS, multiple sclerosis; HC, healthy controls.

The differentially activated brain regions in the NMOSD, MS, and control groups included the bilateral insula, bilateral parahippocampal gyrus, bilateral hippocampus, bilateral superior temporal gyrus, left rectus, left middle frontal gyrus, right precentral gyrus, left orbital inferior frontal gyrus, left putamen, left inferior frontal gyrus, left cuneus, left rolandic operculum, and left paracentral lobule (*P* < 0.05, Figure 2 and Table 2, AlphaSim corrected).

**TABLE 2:** Results of difference analysis of activation in brain regions among groups.

Activated brain region	Brodmann	MNI coordinates			*F-*values	Clusters (voxel)
		X	Y	Z		
Left parahippocampal gyrus	39	−12	−6	−18	5.606	43
Right hippocampus	38	15	−9	−15	6.036	15
left rectus	27	−3	45	−21	7.727	11
Right parahippocampal gyrus	40	17	−33	−6	11.79	16
Left insula	29	−38	14	−6	6.510	162
Left orbital inferior frontal gyrus	15	−38	24	−3	4.085	65
Left inferior frontal gyrus	13	−37	31	6	3.418	34
Left superior temporal gyrus	81	−48	6	−4	4.052	26
Left putamen	73	−22	20	−2	5.081	24
Right hippocampus	37	−18	−24	−9	7.421	19
Right insula	30	39	6	−6	4.980	38
Right superior temporal gyrus	82	69	−12	−6	8.369	63
Left rolandic operculum	17	−42	−9	6	5.203	34
Left middle frontal gyrus	7	−18	42	27	5.786	20
Left cuneus	45	0	−90	27	4.086	17
Right precentral gyrus	2	36	−15	45	4.342	13
Left paracentral lobule	69	−15	−9	66	4.511	35

*MNI, Montreal Neurological Institute.*

**FIGURE 3:**
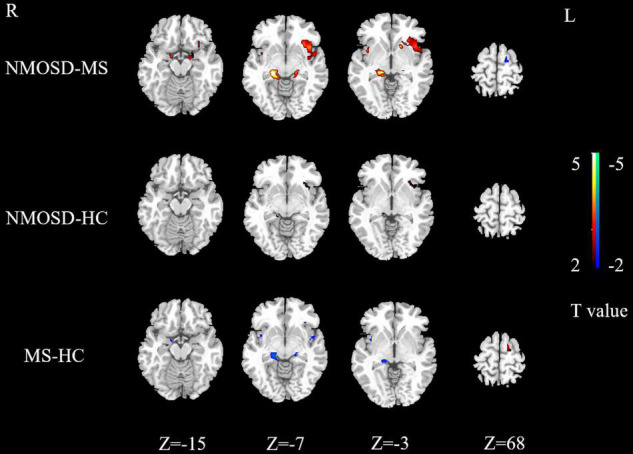
Differences in activated brain regions between NMOSD and MS, NMOSD and HC, MS and HC. Yellow to red indicates strong to weak activation. Blue to green indicates strong to weak activation. NMOSD, neuromyelitis optica spectrum disease; MS, multiple sclerosis; HC, healthy controls.

Activation intensity in the NMOSD group was weaker than the control group in the left rectus, right superior temporal gyrus, and left cuneus. Activation intensity was stronger in the NMOSD than in the control group in the left insula and left middle frontal gyrus (*P* < 0.05, Figure 3).

Activation intensity of the bilateral hippocampus, right parahippocampal gyrus, right insula, left rectus gyrus, and right precentral gyrus in the MS group was weaker than in the controls, while the left paracentral lobule had a stronger activation intensity in the MS group (*P* < 0.05, Figure 3).

Brain regions with weaker activation intensity in the NMOSD group than in the MS group included the left paracentral lobule and right superior temporal gyrus, while those with stronger activation intensity in the NMOSD group included the bilateral insula, bilateral hippocampus, bilateral parahippocampal gyrus, left superior temporal gyrus, left putamen, left inferior orbital gyrus, and left middle frontal gyrus (*P* < 0.05, Figure 3).

## Discussion

Olfactory disorders can be caused by damage in different parts of the olfactory pathway, and the damage in the lumen, superior olfactory, and olfactory nerves is the main cause of damage to the peripheral olfactory pathway. They may also result from damage to the olfactory bulb, olfactory layer, and olfactory system processing areas (Schmidt et al., 2013). Olfactory dysfunction is an early sign of neurodegenerative diseases (Dan et al., 2021). Prospective studies reveal that olfactory dysfunction can predict the occurrence of amnestic mild cognitive impairment and its progression to Alzheimer’s disease (Roberts et al., 2016). Furthermore, it has been adopted as a diagnostic criterion for PD by the International Association for Parkinson’s Disease and Motor Disorders (Postuma et al., 2015). Olfactory dysfunction is an important clinical marker and predictor of these diseases and can help identify disease risk.

Olfactory dysfunction can occur in NMOSD and MS, but the specific mechanism of olfactory dysfunction in patients has not been fully elucidated. Some studies suggest that olfactory function is not only negatively correlated with the damage load in olfactory brain regions (Doty et al., 1997), but is also associated with longitudinal changes in the number of plaques in central olfactory brain regions (Doty et al., 1999), which was also reported by Schmidt et al. (2011) regarding MS. Zhang et al. (2015) observed that the decrease in olfactory function-related gray matter volume was associated with olfactory dysfunction in NMOSD patients. It suggests that atrophy or microdamage of olfactory structures may be responsible for olfactory dysfunction. A study that compared the difference in olfactory function and olfactory-related gray matter volume between NMO and MS patients revealed different regions of gray matter atrophy in both patients. The right orbitofrontal cortex volume in NMO patients with dysosmia was higher than that dysosmia in MS patients. However, the volume of gray matter decreased in the right parahippocampal gyrus and piriform layer in patients with MS (Li et al., 2018), suggesting that the mechanisms of olfactory disorders in NMO and MS may be different.

In this study, the VAS scale was used to assess the olfactory function of patients. The results indicate that the olfactory function of the NMOSD and MS groups was lower than the control group, but this method could only roughly evaluate olfactory function. In this study, an olfactory event-related fMRI design was used to detect significant differences in the activation of brain regions between the NMOSD and MS groups, such as the bilateral insula, left inferior orbital frontal gyrus, bilateral parahippocampal gyrus, bilateral hippocampus, left superior temporal gyrus, left middle frontal gyrus. The activation intensity of patients with NMOSD in the bilateral insula, left orbital inferior frontal gyrus, bilateral parahippocampal gyrus, and bilateral hippocampus was stronger than those in the MS group. The olfactory function may involve neural networks distributed in multiple regions and pathways (Doty et al., 1997). Positron emission tomography (PET) neuroimaging studies reveal that the increase in local cerebral flow in the right orbitofrontal cortex and bilateral insula is related to odor perception, and the left orbitofrontal cortex was also obviously activated during the imagination of odor (Djordjevic et al., 2005). The orbitofrontal cortex and insula cortex are secondary olfactory structures that receive olfactory information transmitted from the primary olfactory cortex. The orbitofrontal cortex is the key structure for olfaction and acts as a nucleus in olfactory processing. It forms a bidirectional connection with the piriform posterior cortex and the amygdala to integrate olfactory information (Gottfried and Zald, 2005; Kjelvik et al., 2012; Howard and Gottfried, 2014).

Some studies have demonstrated that the activation of the piriform and orbitofrontal cortex can also be induced in the presence of odor stimulation during sniffing (Sobel et al., 1998). Activation of the left insula is the most stable during olfactory stimulation (Kurth et al., 2010). At the same time, the insula appears to have significant activity in the process of scent discrimination; it plays a key role in the integration of multiple senses. The dorsolateral prefrontal cortex is the primary structure of the olfactory center, which receives olfactory inputs from various regions such as the orbitofrontal cortex, amygdala, insular cortex, and is involved in odor memory and cognitive function related to olfactory tasks (Karunanayaka et al., 2014).

Studies on olfactory disorders caused by brain injury demonstrate that the olfactory discrimination function of patients with medial temporal lobule or frontal lobule is decreased, but the olfactory threshold is normal and the frontal lobule activation in the process of olfactory discrimination may be related to memory (Plailly et al., 2007). Our results show that the activation of cerebral regions in NMOSD patients differs from healthy adults, including the left insular, left middle frontal gyrus, right superior temporal gyrus, and left cuneus. While the activation of cerebral regions in patients with MS, in regions such as the right insular, bilateral hippocampus, and right parahippocampal gyrus, was different from that in healthy adults. OuYang et al. (2020) confirmed olfactory-related changes in cerebral networks of patients with MS. Using fMRI imaging, we observed changes in olfactory-related brain regions and differences in the activation of olfactory-related brain regions in NMOSD and MS patients, it suggests that the pathological changes of the two diseases may involve olfactory disorders caused by olfactory networks, but there are different olfactory-related brain networks between the two diseases.

## Limitations

This study also has some limitations. First, the olfactory function of patients was roughly evaluated by the VAS score, which cannot fully reflect their precise olfactory status. In our future research efforts, we plan to incorporate Sniffin’ Sticks test or University of Pennsylvania’s smell identification test (UPSIT) to better assess patients’ olfactory function, combined with fMRI to explore the changes that occur in olfactory-related brain regions in different types and degrees of olfactory disorders. Second, the sample size of the NMO and MS groups was relatively small, which may have biased the statistical analyses, and which could not be divided into groups according to the state of their disease, the course of the disease, or the degree of olfactory impairment. Moreover, participants included only patients with NMOSD and MS with decreased olfactory function. Fewer AQP4-IgG-negative NMOSD patients were recruited, and the AQP4-IgG status was not divided into two groups. In our future study, we plan to increase the sample size, conduct a longitudinal study of NMOSD and MS from the two levels of the presence or absence of olfactory disorders, and dynamically observe the changes in olfactory function and olfactory-related brain networks in patients with NMOSD and MS with different disease progression, which will help clarify the pathological mechanism or nuclear networks of olfactory disorders in NMOSD and MS.

## Conclusion

In summary, our results indicate that multiple brain regions are activated during olfactory stimulation in NMOSD and MS patients. Further, there were differences in activation sites and intensity between both patients. The reason for this observation may be that different pathological mechanisms of the two diseases lead to differences in damage to the olfactory cortex and changes in olfactory-related brain networks. The evaluation of the olfactory function of pre-NMOSD and MS is mainly based on subjective olfactory detection and olfactory system structure imaging, but there are few studies on functional magnetic resonance imaging of the olfactory system. This study explored the olfactory function of patients with NMOSD and MS through olfactory event-related design, which has a good spatiotemporal effect, and can expand the study of structural lesions such as olfactory bulb and olfactory bundle related to olfactory disorders to olfactory-related brain networks. Objectively exploring the olfactory function of patients with NMOSD and MS more completely provides a theoretical basis for the study of the mechanism of dysosmia in the future, and may be helpful in providing a new reference index for the clinical differentiation and disease evaluation of NMOSD and MS.

## Data Availability Statement

The original contributions presented in the study are included in the article/supplementary material, further inquiries can be directed to the corresponding author/s.

## Ethics Statement

The studies involving human participants were reviewed and approved by the Ethics Committee of the Affiliated Hospital of North Sichuan Medical College. The patients/participants provided their written informed consent to participate in this study.

## Author Contributions

SH and TP performed all data analysis and wrote the manuscript. SH, TP, WH, and CG were responsible for the acquisition of clinical and MRI data. JT and CH analyzed the MRI data. XW and JT designed the experiment and revised and finalized the manuscript. All authors made significant contributions to the article and approved the final version.

## Conflict of Interest

The authors declare that the research was conducted in the absence of any commercial or financial relationships that could be construed as a potential conflict of interest.

## Publisher’s Note

All claims expressed in this article are solely those of the authors and do not necessarily represent those of their affiliated organizations, or those of the publisher, the editors and the reviewers. Any product that may be evaluated in this article, or claim that may be made by its manufacturer, is not guaranteed or endorsed by the publisher.
